# MiR-200-3p Is Potentially Involved in Cell Cycle Arrest by Regulating Cyclin A during Aestivation in *Apostichopus japonicus*

**DOI:** 10.3390/cells8080843

**Published:** 2019-08-06

**Authors:** Shanshan Wang, Muyan Chen, Yingchao Yin, Kenneth B. Storey

**Affiliations:** 1 Key Laboratory of Mariculture (Ocean University of China), Ministry of Education, Ocean University of China, Qingdao 266003, China; 2 Institute of Biochemistry, Carleton University, Ottawa, ON K1S 5B6, Canada

**Keywords:** cell cycle, miR-200-3p, *AjCA*, *Apostichopus japonicus*, aestivation

## Abstract

The sea cucumber (*Apostichopus japonicus*) has become a good model organism for studying environmentally induced aestivation in marine invertebrates. We hypothesized that mechanisms that arrest energy-expensive cell cycle activity would contribute significantly to establishing the hypometabolic state during aestivation. Cyclin A is a core and particularly interesting cell cycle regulator that functions in both the S phase and in mitosis. In the present study, negative relationships between miR-200-3p and *AjCA* expressions were detected at both the transcriptional and the translational levels during aestivation in *A. japonicus*. Dual-luciferase reporter assays confirmed the targeted location of the miR-200-3p binding site within the *AjCA* gene transcript. Furthermore, gain- and loss-of-function experiments were conducted in vivo with sea cucumbers to verify the interaction between miR-200-3p and *AjCA* in intestine tissue by qRT-PCR and Western blotting. The results show that the overexpression of miR-200-3p mimics suppressed *AjCA* transcript levels and translated protein production, whereas transfection with a miR-200-3p inhibitor enhanced both *AjCA* mRNA and AjCA protein in *A. japonicus* intestine. Our findings suggested a potential mechanism that reversibly arrests cell cycle progression during aestivation, which may center on miR-200-3p inhibitory control over the translation of cyclin A mRNA transcripts.

## 1. Introduction

The sea cucumber, *Apostichopus japonicus*, is an important food and medicinal species with high commercial value that is widely grown and cultured in Asia [1]. This species is a model marine aestivator that endures a prolonged torpor annually (lasting up to 100 days), brought about by elevated sea water temperatures, and is accompanied by a series of typical characteristics of hypometabolism such as metabolic rate depression, organ atrophy, body mass reduction, energy redistribution and immune system modification [2,3]. The period of aestivation shortens the growing cycle of *A. japonicus* and results in economic losses for the sea cucumber aquaculture industry. Research on the mechanisms involved in sea cucumber aestivation has surged in the past two decades and has shown that sea cucumbers have developed various physiological and biochemical strategies to overcome estivation-associated stresses. These strategies facilitate survival over prolonged periods of dormancy, as seen among other aestivators [2,4,5], and, after arousal from aestivation, promote the recovery of all functions back to normal. With the coming of the omics era, the molecular mechanisms underlying the physiological and biochemical strategies that support aestivation are gradually being unraveled. Over numerous studies, we have confirmed that long-term aestivation in sea cucumbers is supported by molecular modifications that include the global suppression of transcription and translation, reversible protein phosphorylation and epigenetic modifications [5,6,7,8,9,10,11]. The types of regulatory mechanisms that are best suited for long-term torpor survival must be easily induced, readily reversed, and have a low energy demand [12]. Since their early recognition [13], microRNAs (miRNAs) have become known as one of the most important post-transcriptional regulatory mechanisms, fulfilling the requirements for a mechanism that could be key to aestivation control. MiRNAs suppress gene expression by binding to gene transcripts and targeting them either for degradation or storage. In situations when animals are responding to extreme environmental stress (e.g., estivation, hibernation, anoxia and freezing) [14], select miRNAs are known to be involved in up- or down-regulating various cellular processes such as apoptosis, cell cycle progression, signal transduction, and lipid metabolism [15,16,17,18,19,20,21]. However, until now, miRNA-targeted regulatory mechanisms supporting aestivation in marine invertebrates have rarely been documented. A prior limited study by our group suggested the potential contribution of miR-200-3p in the control of fatty acid metabolism by regulating the expression of the *AjEHHADH* gene (encoding enoyl-CoA hydratase and 3-hydroxyacyl-CoA dehydrogenase) during aestivation in sea cucumbers [11].

The suppression of energy-expensive cellular processes is the common survival strategy during hypometabolism. For example, the cell cycle, one of the most energy-expensive costs for cells, is typically strongly suppressed or arrested in tissues as one component of overall metabolic depression [22]. It has been reported that cell cycle arrest is crucial for ATP (adenosine-triphosphate) homeostasis and the conservation of fuel stores in hibernating thirteen-lined ground squirrels, *Ictidomys tridecemlineatus* [23], and to achieve long-term anoxia endurance in the turtle *Trachemys scripta elegans* [22]. However, little is known about the epigenetic regulation mechanisms involved in cell cycle arrest operating during hypometabolism, in particular, for aestivation in sea cucumbers.

Cyclin A was the first cyclin identified and subsequently cloned in organisms [24]. Cyclin A is a pivotal regulator of cell cycle progression at the onset of both DNA replication and mitosis [25]. The mRNA and protein products of Cyclin A begin to accumulate in the late G1 stage and peak in the S phase [26]. Many studies have reported a role for miRNA in regulating the proliferative state of human tumor cells by targeting cyclins [27,28,29,30]. However, the potential roles of miRNA in states of hypometabolism where proliferation is suppressed, and the roles of Cyclin A and miRNA-targeted regulatory mechanisms in sea cucumbers remain unknown.

Our present work aimed to study the potential role of miR-200-3p in inhibiting cell proliferation during estivation in the intestine of *A. japonicus* by targeting cyclin A gene expression. We focus on the intestine in the present study because it is the major site responsible for the strong metabolic rate depression seen under deep aestivating conditions, and the global miRNA expression profile of this organ has also been constructed using Solexa deep sequencing technology in our previous study [5]. We initially characterized changes in the transcription and translation of Cyclin A in the intestine of *A. japonicus* over the whole period of aestivation. In vivo and in vitro analyses of the functional relationship between miR-200-3p and Cyclin A were also assessed. The results yielded a picture of the control of Cyclin A in sea cucumber tissues and its adaptive regulation in aestivation-induced post-transcriptional suppression.

## 2. Materials and Methods 

### 2.1. Animals

Adult sea cucumbers (males and females) (*A. japonicus*), 90 ± 10 g, were collected from the coast of Qingdao (Jiaozhou Bay of the Yellow Sea, China). Three stages of *A. japonicus* were sampled and dissected right after capture. Non-aestivating (NA) animals were sampled on 12 May, when the seawater temperature was about 15 °C and sea cucumbers had already recovered fully from aestivation. Animals in deep-aestivation (DA) were sampled on 21 July, when seawater temperature was above 28 °C. These animals were sampled after about 15 days of continuous aestivation as indicated by the cessation of feeding and locomotion, and the degeneration of the intestine into a very thin and small string (about 2 mm). Animals in arousal from aestivation (AA) were sampled on 12 October, when seawater temperature had decreased back to 15 °C. These animals were recently aroused from aestivation, and moving and feeding were observed. For each stage (NA, DA and AA), 10 individuals were sacrificed and intestine tissues were dissected, cleared of contents and flash frozen in liquid nitrogen. All samples were kept at −80 °C for later analysis. The study protocol was approved by the Experimental Animal Ethics Committee of the Ocean University of China.

### 2.2. Prediction of the miR-200-3p Target

TargetScan 5.2 and Miranda 3.3a software was used to predict the gene targets of miR-200-3p action, based on our previous transcriptome and proteome databases for *A. japonicus* [8,9], following the criterion in the seed region—no mismatch between 2–8 nt on the end of miRNA (7mer-m8), and all the parameters were set as default values. Potential targets of miR-200-3p were also predicted using the Miranda toolbox, with parameters set up as follows: single-residue pair score less than a threshold value of 90 and a minimum free energy lower than −10 kcal/mol. Combining these analyses, *cyclin A* was selected as a strong candidate target gene of miR-200-3p.

### 2.3. RNA Extraction, Cyclin A cDNA Cloning and Sequence Analysis

Total RNA was isolated from intestinal tissues of *A. japonicus* using Trizol (TransGen Biotech, Beijing, China, Cat No. J20921) according to the manufacturer’s instructions. Intestinal tissues of *A. japonicus* were ground into powder using liquid nitrogen, and 50 to 100 mg were added to 1.5 mL centrifuge tubes with 1 mL Trizol, then centrifuged at 14,000 rpm at 4 °C for 10 min. The supernatant was moved to a new tube and the Trizol extraction was repeated on the pellet. Then, the second supernatant was mixed with the first and 200 µL chloroform was added in a new centrifuge tube (Sinopharm Chemical Reagent Beijing, Beijing, China, Cat No. 10006818). After mixing for 30 s and resting for 3 min, tubes were then centrifuged at 14,000 rpm at 4 °C for 15 min, and the supernatant was removed. This step was also repeated twice, and then the final supernatants were combined and mixed with the same volume of isopropanol (Sinopharm Chemical Reagent Beijing, Beijing, China, Cat No. 80109218) in a new centrifuge tube at −20 °C for 10 min, then centrifuged at 14,000 rpm at 4 °C for 8 min. Supernatants were discarded and pellets were dried at room temperature for 10 min, followed by addition of 20–50 µL RNase-free Water (Takara, Japan, Cat No. 9012) to dissolve RNA for 30 min. RNA concentration and quality were determined using a NanoDrop 2000 and 1% agarose gel electrophoresis, respectively (Thermo, Waltham, MA, USA).

The full-length cDNA sequences were cloned using a SMARTer^®^ RACE (rapid amplification of cDNA ends) 5′/3′ Kit (Clontech, Mountain View, CA, USA, Cat No. 634858) following the manufacturer’s instructions. The primer sets for *cyclin A* 5′/3′ RACE are listed in Table 1. Polymerase chain reaction (PCR) amplification was carried out using Tks Gflex™ DNA Polymerase (Takara, Kusatsu, Japan, Cat No. R060A) in a volume of 50 µL. PCR was performed under the following conditions: 95 °C for 1 min; 5 cycles of 94 °C for 30 s, 70 °C for 30 s, and 72 °C for 3 min; 5 cycles of 94 °C for 30 s, 68 °C for 30 s, and 72 °C for 3 min; 5 cycles of 94 °C for 30 s, 66 °C for 30 s, and 72 °C for 3 min; 20 cycles of 94 °C for 30 s, 64 °C for 30 s, and 72 °C for 3 min, followed by a final cycle of 72 °C for 10 min. The PCR products were eluted from a 2% agarose gel using the NucleoSpin Gel and PCR Clean-Up Kit (Clontech, Mountain View, CA, USA, Cat No. 740609.10) following the manufacturer’s instructions, then cloned into the pMD19-T vector (Takara, Japan, Cat No. 6013) and transformed into *E.coli* DH5α Competent Cells (Takara, Kusatsu, Japan, Cat No. 9057) following the manufacturer’s instructions. Transformed cells were cultured overnight in Luria-Bertani (LB) agar plates containing 100 µg/mL ampicillin. White clones were selected and cultured in SOC medium (Hopebio, China, Cat No. HBDC002) containing 100 µg/mL ampicillin for 12 h at 37 °C. Positive recombinant clones were sequenced by BGI Genomics (Qingdao, China). The sequences were analyzed and assembled using DNAStar software (DNAStar Inc., USA) to obtain the full-length cDNA and identify the open reading frame (ORF). The deduced amino acid sequence of *AjCA* was analyzed using an on-site program (http://www.bio-soft.net/sms/index.html). The functional sites or domains in the amino acid sequence were predicted using Interpro (http://www.ebi.ac.uk/interpro/) software.

### 2.4. Expression Level Analysis of miR-200-3p and AjCA

Total RNA was isolated as described above from *A. japonicus* intestine tissues of the NA, DA and AA groups, and the good quality of the RNA was indicted by a 260/280 ratio of 1.8~2.1. For qRT-PCR analysis of miR-200-3p, first-strand cDNA was synthesized using the Mir-X miRNA First-Strand Synthesis Kit (Takara, Kusatsu, Japan, Cat No. 638313), and then qRT-PCR was performed using the SYBR^®^ Premix Ex Taq^TM^ II Kit (Takara, Kusatsu, Japan, Cat No. RR820) with the mRQ 3′primer supplied with the kit and miR-200-3p specific primer (Table 1). The 5.8 s rRNA was selected as the internal control (Table 1) [11]. For qRT-PCR analysis of *AjCA*, the first-strand cDNA was synthesized using a PrimeScript™ RT reagent Kit with gDNA Eraser (Takara, Kusatsu, Japan, Cat No. RR047A), and qRT-PCR was performed using the SYBR^®^ Premix Ex Taq^TM^ Kit (Takara, Kusatsu, Japan, Cat No. RR420) with the *AjCA* specific primer pairs (Table 1). β-tubulin (TBB, PIK103) was selected as an internal control and had been previously validated as being unchanged during *A. japonicus* aestivation in an initial study of reference gene stability [31]. β-actin (ACTB, PIK61412.1) was also selected as another internal control, which was mainly to ensure the constant expression of an internal control and made the results more stable and accurate. The expression levels of miR-200-3p and *AjCA* were detected using the StepOnePlus system (ABI Inc., Foster City, CA, USA). Each sample was run in three technical triplicates. Melting-curve analysis of the amplification products was performed at the end of the PCR to confirm specificity. 

Protein extraction and Western blot analysis were performed as previously described [11]. In brief, total protein was extracted from intestine tissues using Cell lysis buffer (Beyotime, Shanghai, China, Cat No. P0013) following the manufacturer’s instructions. Protein samples were boiled for 5 min in SDS sample buffer and then separated on 10% SDS-PAGE gels, followed by transfer to polyvinylidene fluoride (PVDF) membranes (Millipore, Bedford, MA, USA, Cat No. IPVH00010). Then, the membranes were incubated with *AjCA* antibody (1:1000, prepared by GenScript, NanKing, China) or β-tubulin antibody (1:1000, CST, Cat No. 2146S) overnight at 4 °C, after blocking with 5% non-fat milk in TBS-Tween 20 buffer (TBST) for 2 h at 30 °C. After washing with TBST for five times (5 min each), the membranes were incubated with goat anti-rabbit IgG (Immunoglobulin G) labeled with HRP (1:10,000; Cat No. 7074S; CST, Danvers, MA, USA) for 2 h at room temperature. Five more washes with TBST followed, and then bound antibodies were detected with an ECL substrate kit (Beyotime, Shanghai, China, Cat No. P0018) according to the manufacturer’s instructions. 

### 2.5. Dual-Luciferase Reporter Assays

The experiment was conducted as described previously [11]. In brief, both wild-type (WT) and mutant (Mut) segments of the *AjCA* 3’UTR (untranslated region) (about 500 bp before and after the binding sites) were cloned into a pmiR-RB-REPORT^TM^ luciferase reporter vector (Ribobio, Guangzhou, China). The selected primers are listed in Table 1. The 293T cells were plated onto 96-well white plates 24 h before transfection. Plasmids constructed with pmiR-RB-REPORT^TM^ vectors were cotransfected with a control Renilla luciferase plasmid (pRL-CMV) (Promega, Madison, WI, USA). The ratio of experimental plasmid to control plasmid was 5:1. Then vectors with 3′UTR of *AjCA* (WT and Mut) were cotransfected with miR-200-3p mimics or a negative control. Luciferase assays were performed using the Dual-Luciferase Reporter Assay System (Promega, USA). In brief, 48 h after transfection, cell lysates were prepared by incubating with 1× passive lysis buffer for 15 min at room temperature. Cell lysates were transferred to 96-well plates, analyzed using the luciferase dual reporter assay kit (Promega, USA) and measured with a Veritas microplate luminometer (Turner BioSystems, Sunnyvale, CA, USA). An over 30% decrease in the fluorescence signal was considered to be confirmation that *cyclin A* was targeted by miR-200-3p.

### 2.6. Loss and Gain-Functional Analysis of miR-200-3p In Vivo

The miR-200-3p mimics, inhibitor and negative control were synthesized at GenePharma (Shanghai, China) and are shown in Table 1. MiR-200-3p mimics, inhibitor and corresponding negative controls were dissolved into RNase-free water to obtain a working solution of 20 mM. Lipo6000^TM^ Transfection Reagents (Beyotime, China) were used following the manufacturer’s instructions. Aliquots of 300 µL mimics or inhibitor as well as a corresponding negative control were mixed with equal volume of Lipo6000^TM^ Transfection Reagents, then mixed with 2400 µL of PBS (phosphate buffered saline) to serve as the working solution. Twenty-four sea cucumbers at the NA stage (100 ± 10 g) were injected with 100 µL of the miR-200-3p mimics or inhibitor or negative control mixes, and a booster injection was given after 24 h. The treated and control animals were sacrificed, and intestine tissues were collected after a further 24 h and stored in liquid nitrogen for further study.

### 2.7. Statistics

The 2^−^^△△Ct^ method was used to analyze the expression levels of both miR-200-3p and *AjCA* mRNA, and the data were subjected to one-way analysis of variance (ANOVA) followed by a Tukey’s post hoc test using SPSS 17.0 software (Chicago, IL, USA). All results are given as mean ± S.E. (*n* = 5). Western blotting bands were quantified using Image-Pro Plus 6.0 software (Media Cybernetics Inc., Rockville, MD, USA) and were also subjected to one-way analysis of variance (ANOVA) followed by a Tukey’s post hoc test (SPSS 17.0 software, Chicago, IL, USA). Results are given as mean ± S.E. (*n* = 3). Different lowercase letters indicate significant differences from the corresponding control (*p* < 0.05). Dual-luciferase reporter data were subjected to a t-test, and the results are given as mean ± S.E. (*n* = 3). The level of statistical significance is shown as *p* < 0.05 (*) or *p* < 0.01 (**).

## 3. Results

### 3.1. Sequence Characterization of AjCA and Target Identification of miR-200-3p

**Figure 1:**
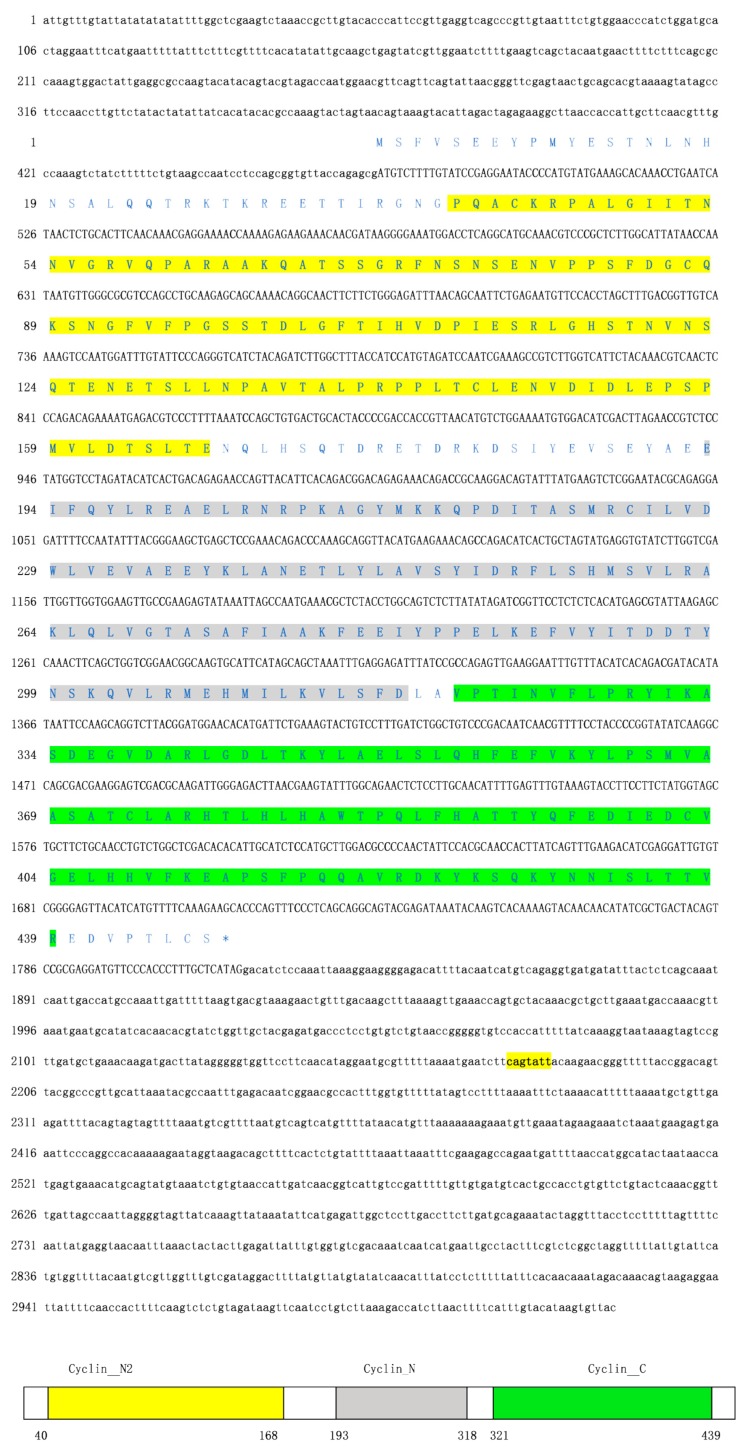
The complete cDNA sequence and deduced amino acid sequence of *AjCA.* Coding and noncoding regions are shown by uppercase and lowercase letters, respectively. The asterisk indicates the translational termination codon. At the bottom of the page is the schematic diagram of domains and characteristic motifs.

**Figure 2:**
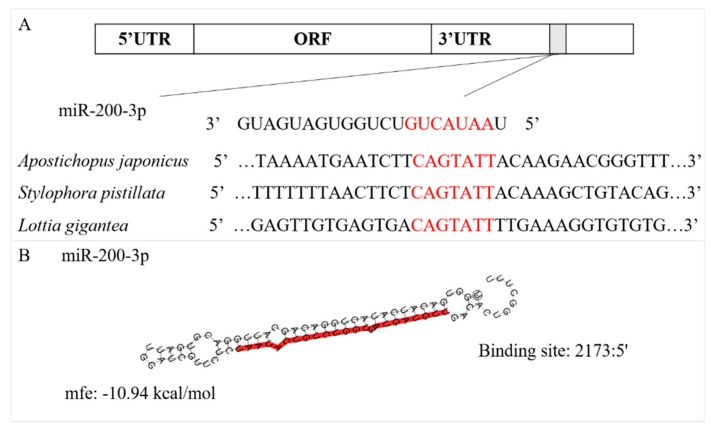
Theoretical binding of miR-200-3p to a conserved region in the 3′UTR of the *AjCA* gene. (**A**) Conservation analysis of the miR-200-3p binding site in the *Cyclin A* gene from the sea cucumber *A. japonicus*, hood coral *Stylophora pistillata* and giant owl limpet *Lottia gigantea*. (**B**) Predicted binding structure of miR-200-3p when binding to the 3’UTR of *AjCA* and the mature miR-200-3p sequence (shown in red), as determined from TargetScan and miRanda programs.

The full-length cDNA of *AjCA* contained 3029 bp, including a 472-bp 5’UTR and a 1,213-bp 3′UTR. The open reading frame (ORF) contained 1344 bp and encoded a polypeptide of 447 amino acid residues with a calculated molecular mass of 50.55 kDa (Figure 1). InterPro analysis indicated that the *AjCA* protein contained Cyclin_N2, Cyclin_N and Cyclin_C domains (Figure 1, Gene accession No. MN055598). Analysis of the putative miR-200-3p binding sites was then performed using TargetScan 5.2 and Miranda 3.3a programs. Results showed that the binding site started from 2,173 bp to 2,179 bp within the 3’UTR of *AjCA*. Importantly, the binding site was identified and found to be conserved in other marine invertebrate groups: the hood coral *Stylophora pistillata* and giant owl limpet *Lottia gigantea* (Figure 2). The theoretical minimum free energy of binding (kcal/mol) was also calculated using the Miranda program. The theoretical prediction of the thermodynamic binding parameters for miRNA 200-3p binding to *AjCA* was calculated to be −10.94 kcal/mol (below the given theoretical parameters −10 kcal/mol), suggesting that *AjCA* is a likely target of miR-200-3p in the sea cucumber (Figure 2).

### 3.2. The Expression of miR-200-3p and AjCA Gene and the Production of AjCA Protein during the Whole Period of Aestivation

**Figure 3:**
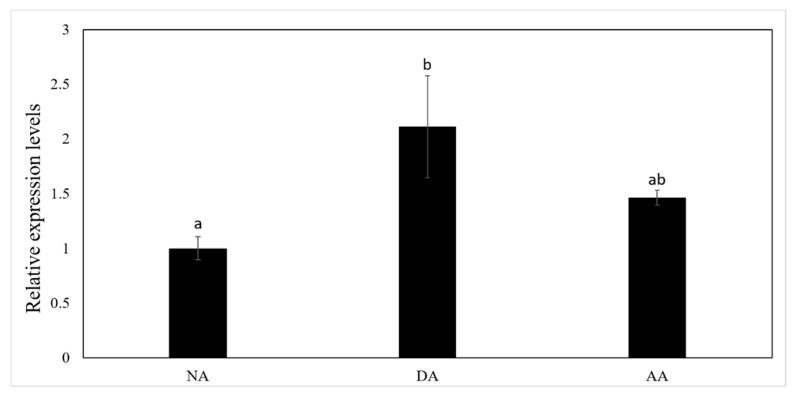
The relative expression of miR-200-3p in the intestine of *A. japonicus* at non-aestivating (NA), deep-aestivation (DA), and arousal from aestivation (AA) stages. The expression of miR-200-3p was detected by qRT-PCR in the intestine of *A. japonicus* from NA, DA and AA groups. Data are means ± SE (*n* = 5 independent trials on tissue from different animals). Different lowercase letters indicate groups that are significantly different from each other (*p* < 0.05).

**Figure 4:**
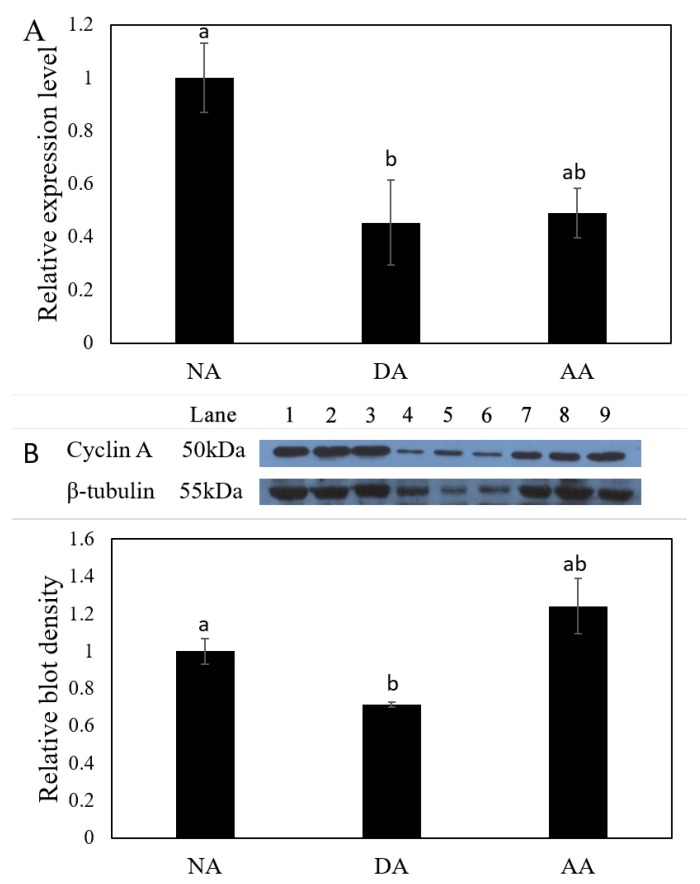
The mRNA expression and protein production levels of *AjCA* in the intestine of *A. japonicus* at NA, DA and AA stages. (**A**) Relative mRNA expression levels of *AjCA* in the intestine of NA, DA and AA groups, determined by qRT-PCR. Values were standardized against β-tubulin and β-actin. Values are means ± SE (*n* = 5). Different lowercase letters indicate groups that are significantly different from each other (*p* < 0.05). (**B**) Relative protein levels of AjCA at the NA, DA and AA stages in intestine as determined by Western blot. Representative bands show blot intensities for NA (lanes 1–3), DA (lanes 4–6) and AA (lanes 7–9) groups. AjCA protein levels were standardized against the corresponding β-tubulin band densities for the same samples. Histograms show the standardized levels for NA, DA and AA. Values are means *AjCA* ± SE (*n* = 3). Different lowercase letters indicate groups that are significantly different from each other (*p* < 0.05).

The expression level of miR-200-3p was significantly up-regulated in the intestine by 2.11 ± 0.47-fold during the DA (deep aestivation) stage as compared with the NA (non-aestivating) stage (*p* < 0.05), but was down-regulated again in the AA (arousal from aestivation) stage (Figure 3). Both the transcript levels of *AjCA* and protein levels of AjCA decreased significantly in intestine at the DA stage to 45 ± 16% and 71 ± 1%, respectively, of the corresponding values during the NA stage (*p* < 0.05). However, in the AA stage (Figure 4A,B), *AjCA* transcript levels remained low (similar to DA), whereas AjCA protein productions rose to a level slightly higher than (but not significantly different from) the NA value. The expression of *AjCA* was negatively correlated with the expression of miR-200-3p in the intestine of *A. japonicus* during aestivation.

### 3.3. Validation of the Interaction between the 3′UTR of AjCA and miR-200-3p by Dual-Luciferase Reporter Assays

**Figure 5:**
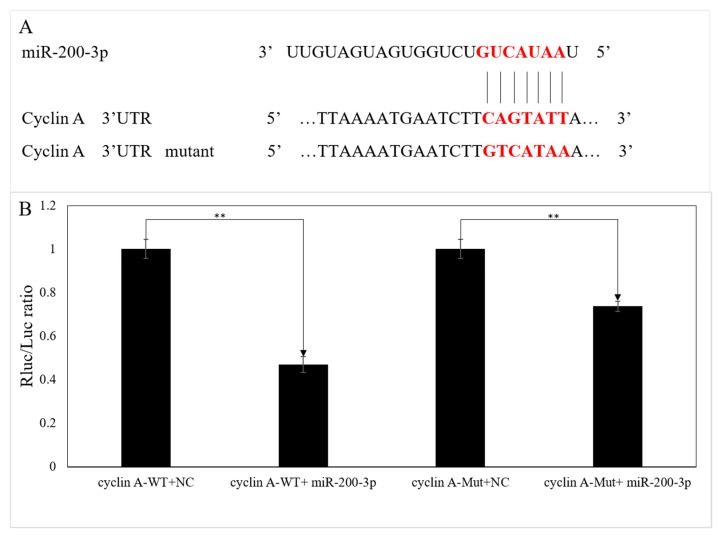
Validation of the binding sites between miR-200-3p and 3′UTR of *AjCA*. (**A**) Schematic representation of the putative miRNA-200-3p targeting sites in *AjCA* mRNA and the respective mutant sites. (**B**) HEK-293T cells were co-transfected with the pmiR-RB-REPORT™ vectors, carrying the wild-type (WT) or the mutated (Mut) *AjCA* 3′-UTR, pRLCMV-Renilla-luciferase, and control miR-200-3p mimics as indicated. ** indicates a significant difference (*p* < 0.01). NC: negative control without miR-200-3p.

The interaction of binding sites on *AjCA* and miR-200-3p was verified by dual-luciferase reporter assays. Information concerning the binding and mutation sites of miR-200-3p in the 3′UTR of *AjCA* is shown in Figure 5A. Compared with the negative control (NC), which lacked co-transfected miRNA, cells with transfected miR-200-3p mimics showed a significant reduction in luciferase reporter activity to 47 ± 4% of the NC value (*p* < 0.01) (Figure 5B), indicating that cells without the co-transfected microRNA were able to express greater amounts of *AjCA* transcripts. Cells containing a mutated (Mut) segment of the *AjCA* 3’UTR also showed a significant, but less pronounced, reduction in luciferase reporter activity, to about 70% of the NC value, suggesting that miR-200-3p was less able to interact with the mutant *AjCA* 3′UTR.

### 3.4. Gain and Loss of Function Analysis of miR-200-3p In Vivo

**Figure 6:**
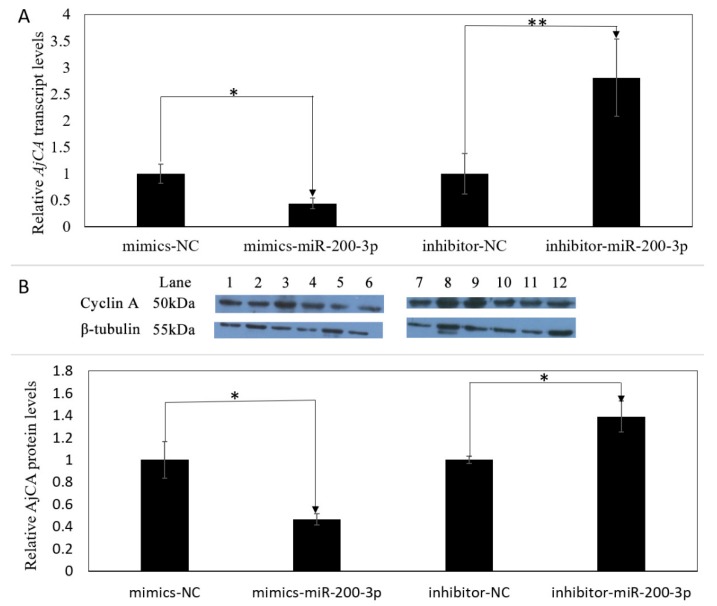
Gain and loss of function analysis of miR-200-3p in the intestine of *A. japonicus* in vivo. (**A**) Relative *AjCA* transcript levels after transfection with miR-200-3p mimics or inhibitor. Values were normalized against β-tubulin and β-actin. Values are means ± SE (*n* = 5). * indicates a significant difference (*p* < 0.05); ** *(p* < 0.01). (**B**) Relative AjCA protein production after transfection with miRNA mimics or inhibitor. Representative bands show blot intensity. Lanes show the treatments as follows: (1–3) miR-200-3p mimics, negative control; (4–6) miR-200-3p mimics; (7–9) miR-200-3p inhibitor, negative control; (10–12) miR-200-3p inhibitor. Corresponding tubulin bands are also shown. Values were standardized against the corresponding densities for β-tubulin. Values are means ± SE (*n* = 3). * indicates a significant difference *(p* < 0.05).

The miR-200-3p targeted regulation of *AjCA* was further verified in vivo using *A. japonicus* samples that were transfected with miR-200-3p mimics, or a miR-200-3p inhibitor, or their negative controls (sequences of all are shown in Table 1). As shown in Figure 6, the overexpression of miR-200-3p mimics significantly decreased both the transcript and protein levels of *AjCA* by about 60% in intestine tissue as compared with the negative control group (*p* < 0.05) (Figure 6A,B). Furthermore, the opposite occurred when the expression of miR-200-3p was inhibited. A significant increase in *AjCA* transcripts of nearly 3-fold occurred, and protein levels increased by about 1.4-fold in *A. japonicus* intestine tissue (*p* < 0.01 and *p* < 0.05, respectively, Figure 6A,B).

## 4. Discussion

The cell cycle is the major process responsible for regulating cell division and tissue growth and is a highly resource- and energy-expensive process [32]. The regulation of the cycle depends on the sequential expression of key cyclins during different phases of the cycle. Cyclin A is a core and particularly interesting regulator that activates two different cyclin-dependent kinases (Cdk1 and Cdk2) and functions in both the S phase and in mitosis [33]. It has been reported that inactivation of cyclin A in Drosophila or mammalian cultured cells arrests the cell cycle in the G2 phase [34]. Cell cycle suppression/arrest is known to be part of hypometabolism in animals that use dormancy or torpor to survive during times of extended environmental stress. Control over cyclins occurs at transcriptional and translational levels as well as by epigenetic mechanisms, such as control over cyclin D1 during anoxia exposure in turtles by microRNA inhibition of mRNA translation [35] and cyclin B arrest by DNA methylation in aestivating sea cucumbers [10]. In our present study, the expression patterns of *AjCA* transcripts and AjCA protein were tracked over the aestivation-arousal cycle. Based on this, we hypothesized that one or more specific control mechanisms, focused on *AjCA*, may probably be central to global cell cycle arrest during sea cucumber aestivation. 

Research over the last decade has demonstrated that miRNAs play integral roles in metabolic rate depression in multiple animal systems, where they help to modulate the flow of mRNA transcripts to one of three fates: immediate availability for translation, storage for later translation, or degradation. By modulating these choices, miRNA action can be a powerful, yet metabolically simple, means of altering global energy expenditure on protein synthesis that is readily adaptable for use in coordinating reversible transitions to and from hypometabolic states. The miR-200 family is one of the best-known miRNA families in mammals [36]. As a member of this family, miR-200-3p has been reported to play roles in cell proliferation, apoptosis and tumorigenesis [37,38]. However, focused studies of miR-200-3p effects within metabolically or environmentally stressed cells are still very limited. In our present study, *AjCA* was putatively identified as a novel target of miR-200-3p using bioinformatics analysis combining TargetScan 5.2 and Miranda 3.3a software, and a negative relationship between miR-200-3p and *AjCA* expression at both transcriptional and translational levels was documented. This correlation suggests that miRNA-200-3p interacted with *AjCA* transcripts at a post-transcriptional level—i.e., diverting transcripts away from the transcriptional apparatus and into degradation or perhaps into storage granules or P-bodies during aestivation. This led to reduced synthesis of AjCA protein. In the deep aestivation (DA) situation, the above effects may also be integrated with other controls that operate at a global level to inhibit overall transcription and translation of all cell cycle proteins during torpor. This is probably needed in the intestine due to its high proliferative potential that must be strongly suppressed during the months of nonfeeding [39]. Therefore, we further investigated miR-200-3p regulation of *AjCA*. Using dual-luciferase reporter assays, the results confirmed that the binding site predicted between miR-200-3p and *AjCA* is the potential targeted location. This binding site was also conserved in other marine invertebrate groups (e.g., hood coral *Stylophora pistillata* and giant owl limpet *Lottia gigantea*), suggesting that *cyclin A* is probably a common gene target of miR-200-3p in marine invertebrates. 

To further support our claims, gain- and loss-of-function experiments were conducted in vivo to verify the interaction between miR-200-3p and *AjCA* by qRT-PCR and Western blotting. The results showed that overexpression of miR-200-3p negatively influenced *AjCA* expression at both at the transcriptional and translational levels in *A. japonicus* intestine. Oppositely, inhibition of miR-200-3p led to increased levels of *AjCA* transcripts and AjCA protein. Hence, we propose that miR-200-3p acts as a regulatory mechanism affecting cyclin A and may also affect other cell cycle components when sea cucumbers enter aestivation. Indeed, the inhibition of cyclin A has been confirmed as one of the conserved and general strategies of environmental stress-induced hypometabolism in other species [16]. 

In summary, this study provides the first demonstration of *AjCA* regulation in aestivating sea cucumbers, *A. japonicus*, and suggests that aestivation-responsive suppression of cyclin A transcripts and protein in the intestine are linked to the actions of microRNA. Additionally, miR-200-3p likely has an influence on the overall proliferation state of cells during hypometabolism, as it is also known to target other genes involved in cell proliferation and apoptosis, such as the FOXO (forkhead box) transcription factor and its related signaling pathways [40]. Further studies are required to assess the full degree of influence exerted by miR-200-3p on both cyclin A and other gene targets in sea cucumbers and their involvement in aestivation and environmental stress resistance. To date, this is difficult since very few functional studies of miRNAs have been conducted among marine invertebrates. However, the characteristics of microRNA regulation dovetail with the need for mechanisms of environmental stress response to be broadly applicable, readily coordinated, easily induced and readily reversed [41]. We hope our studies will push forward this research field and greatly improve our understanding of this epigenetic mechanism in marine invertebrates.

## Figures and Tables

**Table 1:** Primer sequences information.

	Name	Primer Sequences (5′–3′)	Location
RACE	*AjCA*-F1	AGCTGGTCGGAACGGCAAGTGCA	1269–1291
	*AjCA*-R1	GATTGTCGGGACAGCCAGATCAAAGGAC	1414–1441
qRT-PCR	miR-200-3p-F	TAATACTGTCTGGTGATGATG	
	miR-200-3p-R	mRQ 3′primer	
	5.8s-F	ATCACTCGGCTCGTGCGTC	
	5.8s-R	GCCATTTGCGTTCGAATAAGT	
	*AjCA*-RT-F	TATCAAGGCCAGCGACGAAGGAG	1461–1448
	*AjCA*-RT-R	GGAGATGCAATGTGTGTCGAGCC	1594–1616
	*β-actin*-F	AAGGTTATGCTCTTCCTCACGCT	
	*β-actin*-R	GATGTCACGGACGATTTCACG	
	*β-Tubulin*-F	GAAAGCCTTACGACGGAACA	
	*β-Tubulin*-R	CACCACGTGGACTCAAAATG	
In vivo	miR-200-3p mimics-F	UAAUACUGUCUGGUGAUGAUG	
	miR-200-3p mimics-R	UCAUCACCAGACAGUAUUAUU	
	miR-200-3p mimics-NC-F	GAGAUGUUCAAUCGGGUAUUU	
	miR-200-3p mimics-NC-R	AUACCCGAUUGAACAUCUCUU	
	miR-200-3p inhibitor	CAUCAUCACCAGACAGUAUUA	
	miR-200-3p inhibitor-NC	GAAUUACAUGCACCACUCAAU	
Dual-luciferase	*AjCA*-WT-F	GCGGCTCGAGGACATCTCCAAATTAAAGGA	1806–1836
	*AjCA*-WT-R	AATGCGGCCGCGTAACACTTATGTACAAATG	3010–3029
	*AjCA*-MUT-F	TGAATCTTGTCATAAACAAGAACGGGTTTTTAC	2164–2197
	*AjCA*-MUT-R	GTTCTTGTTTATGACAAGATTCATTTTAAAAAC	2155–2187
